# Global assessment of environment, health and economic impact of the novel coronavirus (COVID-19)

**DOI:** 10.1007/s10668-020-00801-2

**Published:** 2020-06-05

**Authors:** Samuel Asumadu Sarkodie, Phebe Asantewaa Owusu

**Affiliations:** Nord University Business School, Bodø, Norway

**Keywords:** COVID-19, Novel coronavirus, Sars-Cov-2, Environmental impact of COVID-19, Economic impact of COVID-19, Health impact of COVID-19, Social distancing measures

## Abstract

The institution of social distancing and punitive measures to contain the spread of COVID-19 through human-to-human transmission has environmental, health and economic impact. While the global pandemic has led to the enhancement of the health system and decline of emissions, economic development appears deteriorating. Here, we present the global environmental, health and economic dimension of the effect of COVID-19 using qualitative and empirical assessments. We report the health system policies, environmental sustainability issues, and fiscal, monetary and exchange rate measures introduced during lockdown across countries. While air pollution is reported to have declined, municipal and medical waste is increasing. The COVID-19 global pandemic uncertainty ranks the UK as the country with the highest uncertainty level among 143 countries. The USA has introduced 100% of pre-COVID-19 crisis level GDP, the highest policy cut-rate among 162 countries. Science, innovation, research and development underpin COVID-19 containment measures implemented across countries. Our study demonstrates the need for future research to focus on environment-health-economic nexus—a trilemma that has a potential trade-off.

## Introduction

The emergence of the novel coronavirus (COVID-19) as a global pandemic has triggered the necessity of environment-health-economic nexus. Hence, the outbreak of COVID-19 pandemic is a public health concern with dire health, environmental and economic consequences (Wang et al. 2020). The first confirmed case of the novel coronavirus, which then appeared like “*pneumonia with unknown aetiology*” was officially reported to the World Health Organization (WHO) on December 31, 2020 (WHO 2020b). The outbreak which initially occurred in a seafood market in Wuhan, China spread across countries through human-to-human transmission and community spread within a short period (Sarkodie and Owusu 2020; WHO 2020c). On March 11, 2020, WHO declared COVID-19 as a global pandemic after the infectious disease spread across 114 countries with 118,000 confirmed cases and 4291 deaths (WHO 2020e). As of April 25, 2020, there were 2,896,746 (~ 377 per million people) reported cases around the globe—of which 202,846 (~ 26 per million people) deaths were recorded alongside 1,993,780 (~ 260 per million people) active cases and 816,685 (~ 106 per million people) recovered cases (Lauren, 2020). Currently, the USA has the highest number of confirmed cases (938,154) and deaths (53,755) among 183 countries, followed by Spain (223,759 confirmed cases and 22,902 deaths), Italy (195,351 confirmed cases and 26,384 deaths), France (160,292 confirmed cases and 22,614 deaths), Germany (156,513 confirmed cases and 5877 deaths) and the UK (148,377 confirmed cases and 20,319 deaths) (Lauren, 2020). Surprisingly, this is the first time in history that coronavirus has triggered a global pandemic; thus, COVID-19 is a public health concern that poses grave threats to health, environment and economic outcomes.

**Fig. 1:**
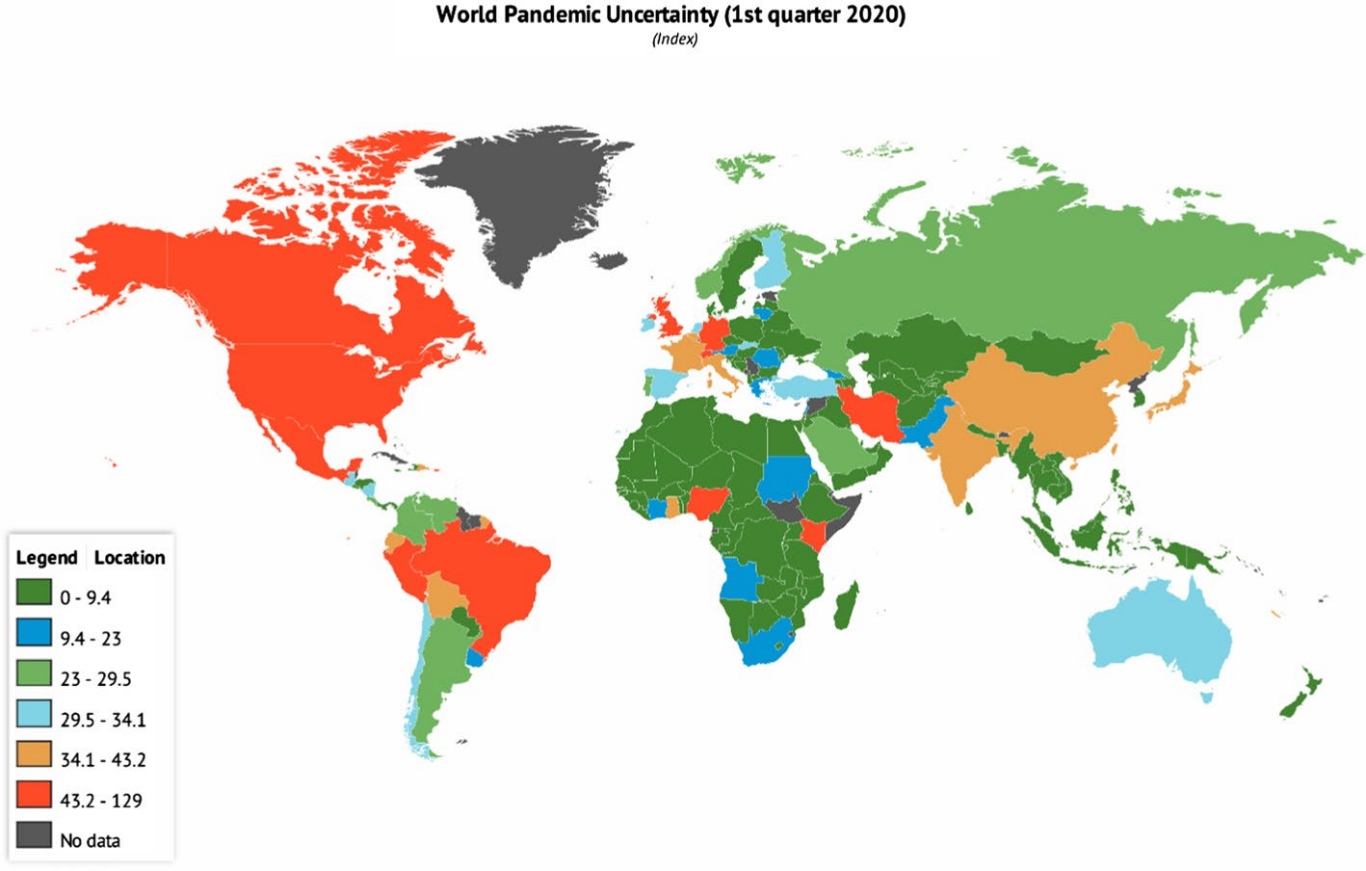
Global Uncertainty Index of COVID-19 pandemic

The novel coronavirus is reported to have attracted more global discussion and pandemic uncertainty compared to SARS (2002–2003), Avian flu (2003–2009), Swine flu (2009–2010) and Ebola (2014–2016) since 1996 (Ahir et al. 2018). Though uncertainty levels due to pandemic are reported to be high in developing countries due to its strong relationship with market volatility and economic uncertainty (Ahir et al. 2018). However, a first-quarter report (Fig. 1) on COVID-19 global pandemic uncertainty^1^ ranks the UK (128.36 index) as the country with the highest uncertainty level among 143 countries towards COVID-19 pandemic. Other countries include Switzerland (91.73), Mexico (67.56), Brazil (66.83), Nigeria (64.27), Canada (61.30), Peru (49.83), Kenya (45.06), Germany (44.91) and the USA (43.57) (Knoema 2020).

During this period of the global pandemic, several measures have been put in place to contain the spread of COVID-19 (Gautam and Hens 2020). Such containment measures include quarantine, travel ban and restrictions, social distance enforcement and lockdown—closure of public places and cancellation of public events. These containment measures put in place to reduce health outcomes of the global pandemic have affected environmental sustainability and economic development. While some studies have reported the environmental impact of COVID-19 (Gautam and Trivedi 2020), no study has yet reported the health and economic implications of the global pandemic. Given this, we present the pros and cons of environmental, health and economic impacts of COVID-19 across countries. We contribute to the existing studies on COVID-19 pandemic by examining the impact of COVID-19 on health outcomes and provide recent policies to improve health quality across the globe. Second, we present the several fiscal measures, monetary policies and private sector economic burden-sharing across countries due to the impact of the social distancing measures on economic sectors. This is essential to understand the global picture of the COVID-19 pandemic from socio-economic and environmental dimensions.

## Results and discussion

Due to the limitation of data and the newness of COVID-19, this study relies on both quantitative and qualitative data from verified sources such as WHO, OECD, COVID Economics and John Hopkins University. We further use the realtime data to construct geographical maps to support the study. Using descriptive statistical tools and collation of information from reliable news portals, we examine and report key environment, health and economic activities since the emergence of the global pandemic.

### Environmental impact

Social distancing policies instituted across countries are reported to have yielded environment sustainability. Total lockdown in many countries saw a halt in carbon and energy-intensive economic sectors such as manufacturing and transportation. The interruption of road transportation and airlines saw overwhelming decongestion in urban centres and traffic level leading to a decline in anthropogenic emissions (Verisk 2020). It is reported that the social distancing measures led to a decline of energy demand and industrial output, hence, affecting environmental quality. Coal-fired power generation is reported to have declined by 50% in China whereas oil consumption declined by 20–30% (Carbon Brief 2020). The reduction in coal and oil consumption led to a decline in carbon dioxide emissions by 25% (100MtCO_2_), corresponding to a 6% reduction in global emissions (Carbon Brief 2020). NASA satellite images showed a reduction in nitrogen oxide emissions by 70% due to a reduction in the consumption of fossil fuels during the lockdown period (UCAR, 2020). Ambient particulate matter (PM) 2.5 is reported to have declined within the short period when social distancing was enforced in Asian and European countries (Gautam 2020; Kasha 2020). Carbon monoxide and aerosol concentration levels are observed to have declined in urban and industrial areas with high economic activities (Gautam and Trivedi 2020; Holthaus 2020). However, no evidence exists regarding the impact of cremation of COVID-19 death victims on sulphur dioxide emissions (Kasha 2020). It is reported that exposure to ambient air pollution increases morbidity and mortality (Sarkodie et al. 2019). People with underlying conditions such as cardiovascular and pulmonary diseases are more susceptible to COVID-19. Meaning that the reduction in air pollution, waterpipe and tobacco use reduces the risk of COVID-19 (WHO 2020d).

There are reports of medical and municipal waste generation linked to the COVID-19 pandemic (ACRPLUS 2020). As much as six times of medical waste (240 metric tonnes) were generated daily in hospitals in China (Calma 2020). Hence, China had to construct new medical waste disposal centres and mobile waste facilities to increase the waste disposal treatment from 50 tonnes to 263 tonnes daily (Mandy 2020; Zhong 2020).

Social distancing and shutdown of the tourism industry are likely to improve biodiversity and enhance the regenerative capacity of the marine habitat (fishing ground) and forest reserve. The reduction in road and railway transportation, and shutdown of commercial, power plants and manufacturing activities are likely to reduce ambient noise levels (Zambrano-Monserrate et al. 2020).

We note that a temporary reduction in atmospheric emissions and environmental degradation in the time of global pandemic does not translate into total environmental sustainability, but a transitory period that will experience a rebound effect during post-COVID-19. The premise of the argument is centred on the grave economic damage caused by the observation of social distancing measures to mitigate the spread of COVID-19. The introduction of welfare cost, health system policies, fiscal, monetary and exchange rate measures are gradually leading many economies into recession. Hence, many economies, especially developing countries, will utilize the scale effect approach — where resource depletion, carbon and energy-intensive economic structure are given priority over environmental sustainability (Sarkodie and Strezov 2018). However, it is too early to conclude on the impact of the aftermath of COVID-19 on environmental pollution.

### Health impact

The strategic objectives of WHO to combat the global pandemic include the interruption of human-to-human transmission, prevent secondary infections and animal transmission and decline cross-boundary spread (WHO 2020b). Other core objectives include the reduction in socio-economic impact via multi-sectoral partnership, treatment opportunities, enhanced diagnostics, therapeutics and vaccines (WHO 2020a).

Several measures to support and significantly impact the health system have been instituted across countries to provide an immediate response to the COVID-19 pandemic.

For example, in Albania, 20 million Euros was provided to procure medical equipment and support medical staff. Frontline nurses, doctors and other health workers received 1000 Euros as additional payment (IMF 2020). Besides, emergency law was instituted to fine private hospitals refusing to provide healthcare assistance with a tune of 40,000 Euros and 83,000 Euros fine to trade entities that breach health safety measures to contain the spread (Gjergj 2020).

Retired and unemployed health care professionals and technicians were recruited in Angola to strengthen and enhance the health system human capacity to provide treatment to affected and quarantined cases (OECD 2020b).

The Brazilian government budgeted 0.4% of GDP for the healthcare system and zeroed taxes and import duties on healthcare-related goods and services (IMF 2020). Ten million rapid test kits were distributed while 5800 employments were offered to doctors with an additional 20% bonus to resident doctors. 2000 beds and 6500 ventilators were hired for intensive care units and telemedicine and online consultations were initiated (UOL 2020).

In China, over 42,000 medical professionals were sent to Wuhan, where the outbreak began (Islamuddin 2020). Two new temporary hospitals were constructed whereas dozens of laboratories were equipped for rapid testing. Medical and pharmaceutical-related goods and services for COVID-19 were exempted from fees whereas medical-related research into vaccines to combat the virus were supported (OECD 2020b).

In Dominican Republic, the government instituted no cost of testing COVID-19 for people older than 59 years, people with 2 or more health-related symptoms, and those with weak health conditions. Two hospitals were designated for solely receiving and treating COVID-19 cases while isolation centres were created in 15 health centres (Squire Patton Boggs 2020).

In terms of health policy enhancement, there has been an acceleration in research and development for vaccines and treatment across countries (OECD 2020b; OECD Health Division 2020). While there is widespread adoption of telemedicine and modern surveillance and tracking systems, mobilization and protection of healthcare professional have been improved (AHPRA 2020). Protection of the aged has been enhanced through the improvement in availability, accessibility and affordability of diagnostic and treatments (GOV.UK 2020). Hospital beds and spaces have been optimized for COVID-19 diagnosis and treatment (SORA 2020). Importantly, medical supplies of ventilators, personal protective equipment, diagnostic tests, and essential medicines have increased across countries (OECD Health Division 2020).

Policies on Science and innovation have played a critical role in responding and containing the spread of COVID-19. Is for a fact that the global pandemic has enhanced the health system and improved science, innovation and health research policy.

### Economic impact

The intensity of the global pandemic (COVID-19) has affected global economic development, resulting in several fiscal measures, monetary policies and private sector economic burden-sharing across countries. The average estimate of economic policy response comprises of monetary stimulus package (% of GDP), fiscal policy package (% of GDP), monetary intervention to control the balance of payment (BOP) and exchange rate (% of GDP), policy rate cut (as a % of pre-crisis level) and total economic stimulus (% of GDP) (Elgin et al. 2020). The economic response policy was used for social intervention programs such as social assistance, social insurance and support labour markets (Gentilini et al. 2020).

**Fig. 2:**
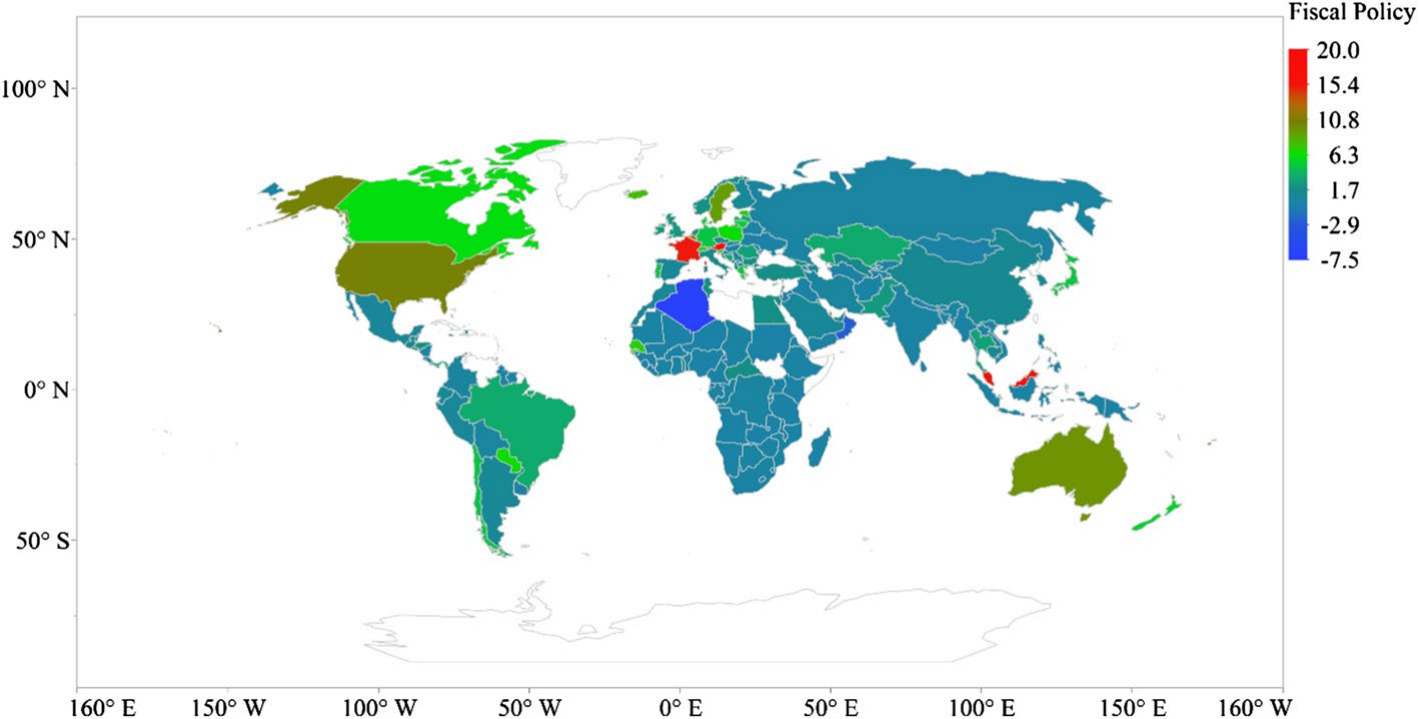
Global distribution of fiscal policy package due to pandemic (% of GDP)

**Fig. 3:**
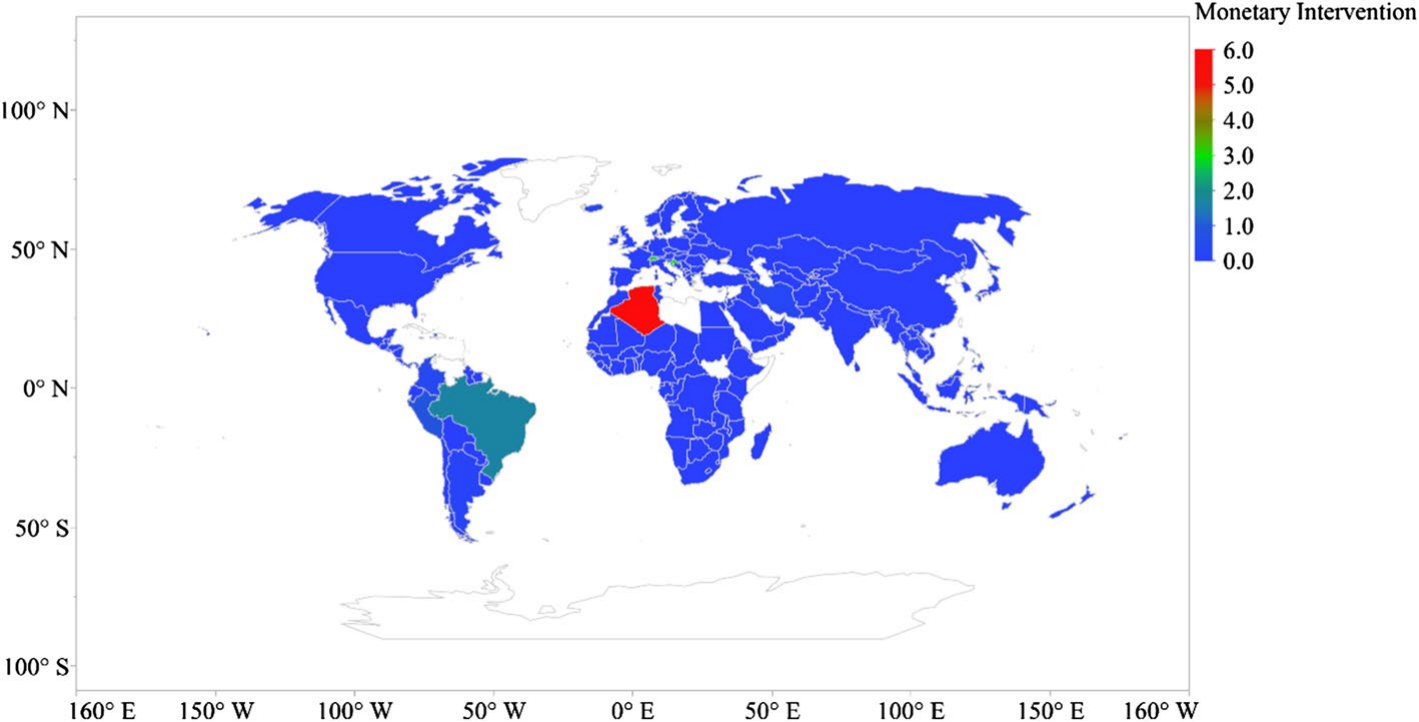
Global distribution of monetary intervention to control BOP and exchange rate due to pandemic (% of GDP)

The highest fiscal policy package presented in Fig. 2 occurs in Austria (~ 17.80% of GDP) across 162 countries, followed by Malaysia (~ 16.22% of GDP), Luxembourg (~ 15.60% of GDP), France (~ 15.30% of GDP), Qatar (~ 13.00% of GDP), Belgium (~ 12.30% of GDP), Malta (~ 12.30% of GDP), the USA (~ 10.50% of GDP), Singapore (~ 10.50% of GDP) and Australia (~ 9.70% of GDP). The highest monetary intervention to control BOP and exchange rate occurs in Algeria (6% of GDP) among 162 countries, trailed by Croatia (2.94% of GDP), Switzerland (2.90% of GDP), Brazil (1.69% of GDP) and Peru (0.90% of GDP) [see Fig. 3].

**Fig. 4:**
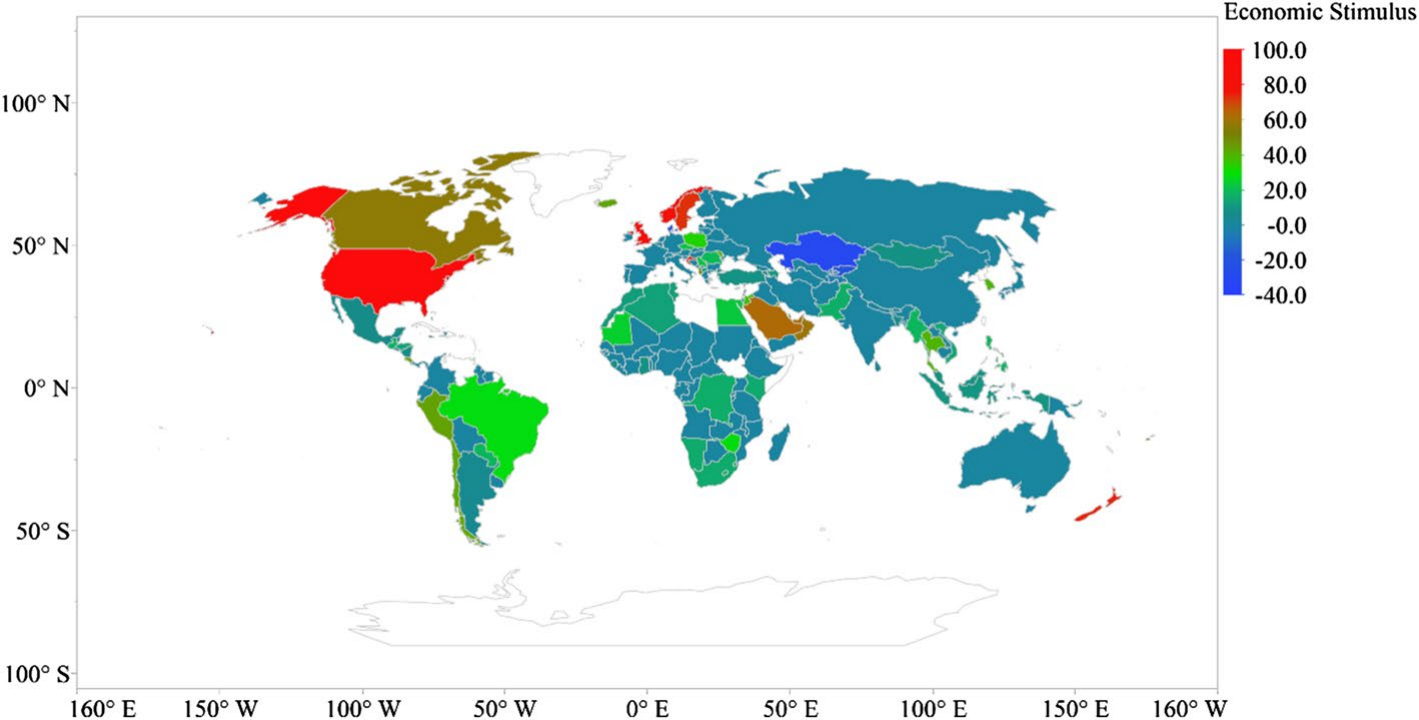
Global distribution of total economic stimulus to pandemic (% of GDP)

The highest total economic stimulus occurs in Bahrain (31.30% of GDP) across 162 countries, followed by Malta (25.61% of GDP), Austria (25.11% of GDP), Luxembourg (22.91% of GDP), France (22.59% of GDP), Oman (22.59% of GDP), Belgium (19.61% of GDP), Sweden (18.65% of GDP), Germany (17.29% of GDP) and Malaysia (16.42% of GDP) [see Fig. 4].

**Fig. 5:**
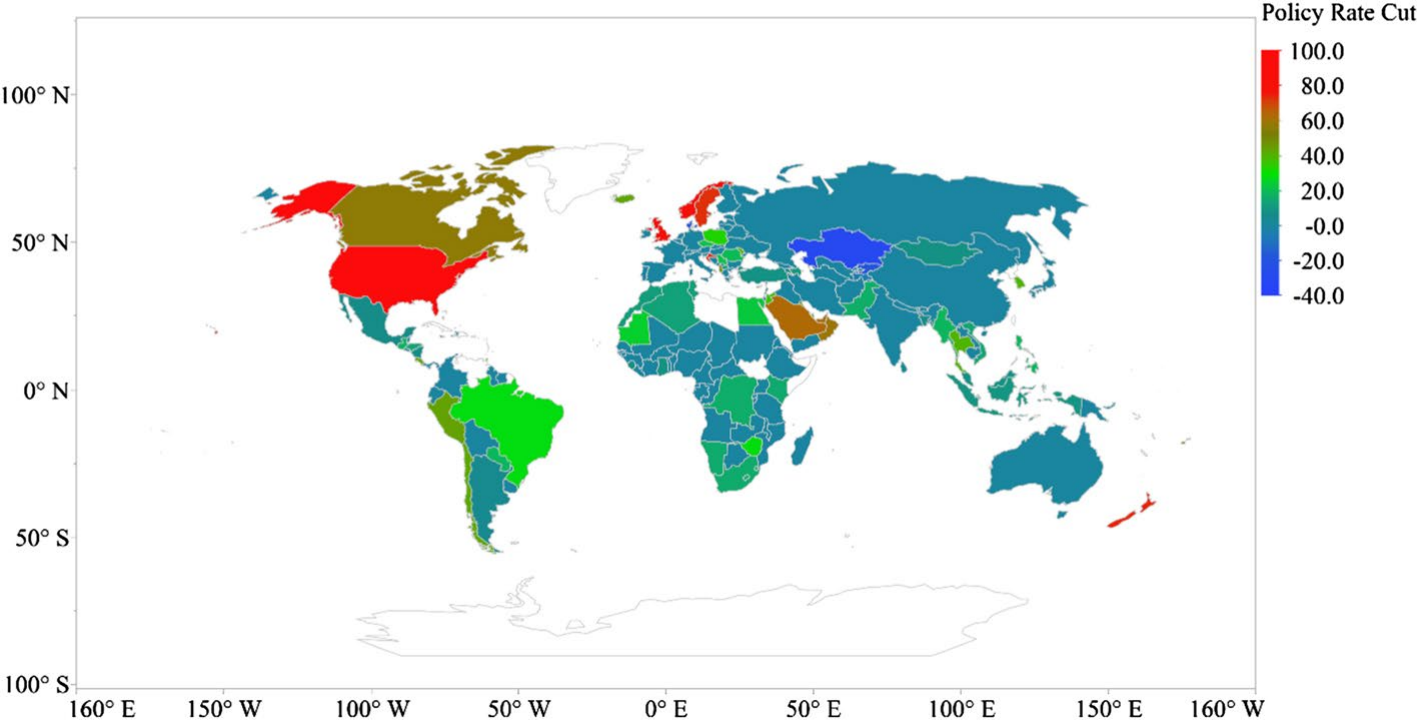
Global distribution of policy rate cut due to COVID-19 (% of pre-COVID-19 crisis level)

The highest reported policy rate cut occurs in the USA (100% of pre-COVID-19 crisis level), the UK (86.67% of pre-crisis level), Norway (83.33%), Croatia (75% of pre-crisis level), New Zealand (75% of pre-crisis level), Sweden (73.33% of pre-crisis level), Saudi Arabia (63.49% of pre-crisis level), the UAE (62.50% of pre-crisis level), Oman (60% of pre-crisis level) and Canada (57.14% of pre-crisis level) (see Fig. 5).

**Fig. 6:**
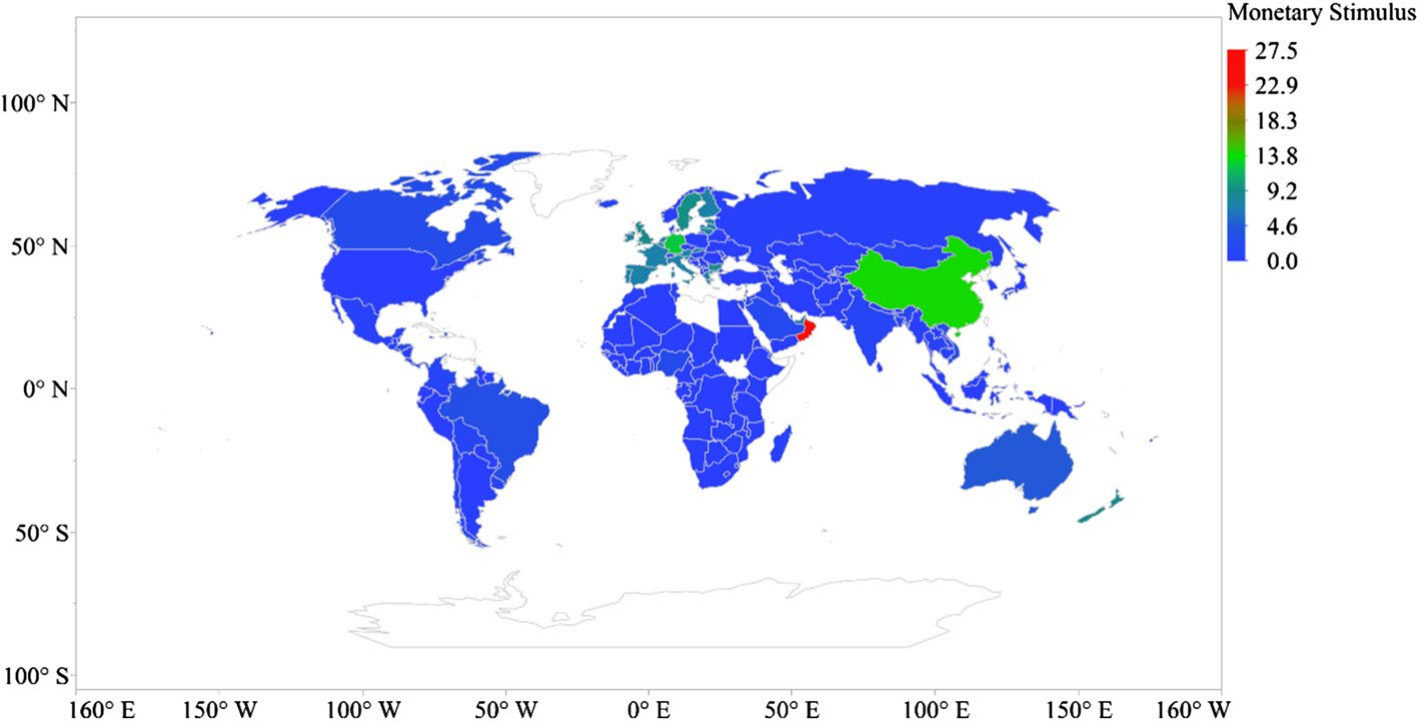
Global distribution of monetary stimulus package to COVID-19 (% of GDP)

The highest monetary stimulus package occurs in Bahrain (26% of GDP) across 162 countries, followed by Oman (25.09% of GDP), China (14.14% of GDP), Malta (13.31% of GDP), Germany (12.49% of GDP), Sweden (9.45% of GDP), the UK 9.09% of GDP), New Zealand (8.86% of GDP), Bulgaria (8.60% of GDP) and Cyprus (7.77% of GDP) (see Fig. 6).

**Fig. 7:**
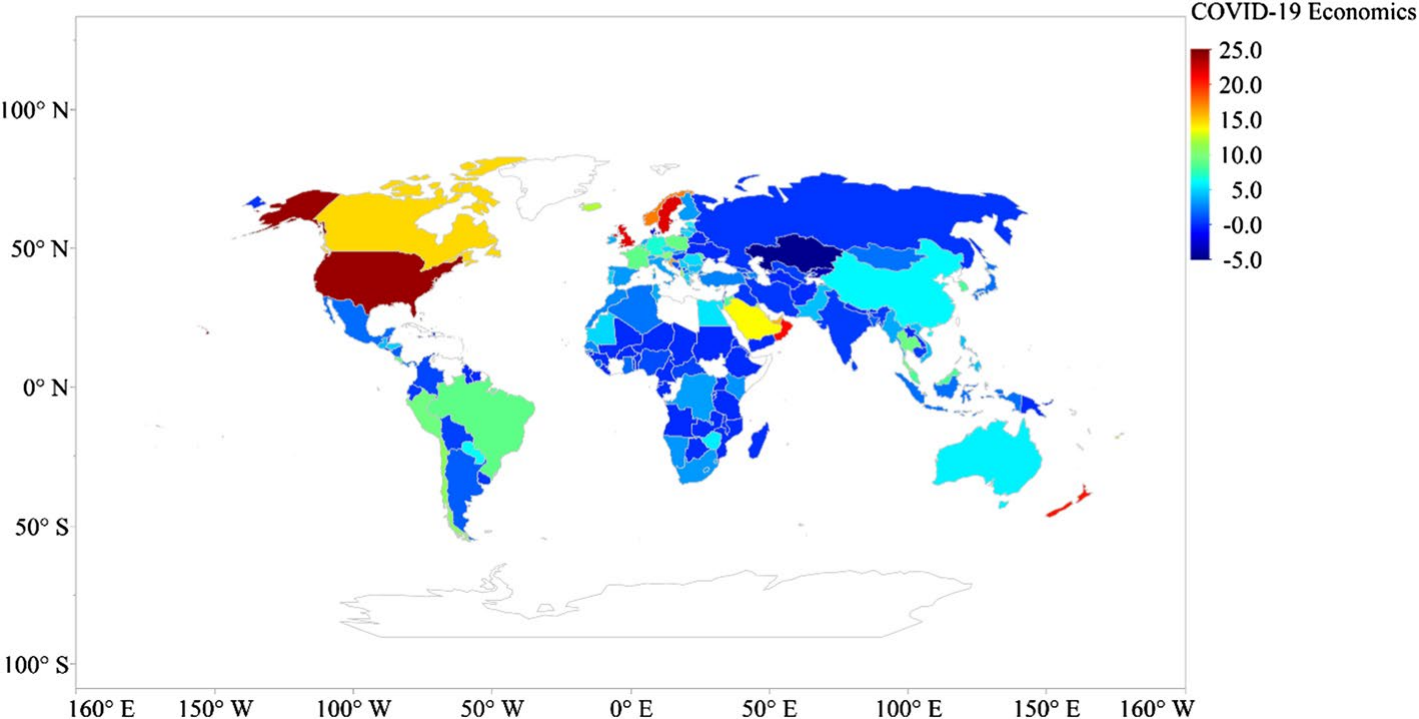
Global average distribution of economic policy response to COVID-19 (% of GDP)

The global distribution of economic policy response to COVID-19 across 166 countries is presented in Fig. 7. The overall average economic policy response to COVID-19 reported in Fig. 7 positions the USA on top, followed closely by Sweden, the UK, Oman and New Zealand. Countries with limited or no economic policy include Kazakhstan, Kyrgyzstan, Denmark, Belarus, Ukraine, Turkmenistan, Yemen, Liberia, Guinea and Laos.

In OECD countries, economic policy responses to COVID-19 amidst containment measures include 100% financial support to affected local firms, ~ 94% income support to self-employed people and residents with loss of job or income, ~ 80% financial assistance to pay rent, mortgages or utilities and ~ 47 to 67% income support to quarantined and sick workers (OECD 2020c). In Austria, ~ 38 billion Euros have been budgeted as emergency funds to cushion COVID-19 affected industries, and tax deferrals for personal and corporate income taxes (IMF 2020). Aside stimulus package and fiscal measures, RM 10 billion was announced by the Malaysian government to support small- and medium-scale enterprises, electricity discount of 15% to support the tourism sector, and 2% discount for various users from the domestic, commercial and industrial sector (PMO 2020). Similarly, ~ 136 million USD (50 billion Naira) credit facility has been budgeted for small- and medium-scale enterprises in Nigeria (CBN 2020). The Norwegian government announced loan scheme valued at NOK 174 billion for the aviation industry, companies, students and vulnerable groups (OECD 2020a). In Russia, 6.11 billion USD (500 billion roubles) was budgeted to cushion regional budgets, firms and households (OECD 2020a). In the USA, an amount of 2 trillion USD has been budgeted for the coronavirus aid, relief and economic security Act, alongside US$ 8.3 billion spending bill and another proposed amount of US$ 108 billion (Heritage 2020; OECD 2020a). Tunisia received about 13 million Euros (TND 40 million) from the World Bank to assist in the fight against the global pandemic. Besides, Tunisia received an IMF emergency assistance loan of 745 million USD to cushion businesses and provide adequate resources for the health sector (IMF 2020). In Saudi Arabia, a 32 million USD stimulus was announced to support economic sectors hit by COVID-19. The debt ceiling was increased from 30 to 50% of GDP whereas the fiscal debt is expected to increase from the projected 6.4% of GDP to 9% (Gulf Today 2020).

It is reported that the choice of a government to undertake strong fiscal stimulus depends mainly on sovereign credit ratings and economic stringency to salvage the deteriorating economic effect of social distancing policies to reduce the spread of COVID-19 (Balajee et al. 2020).

## Conclusion

The critical assessment presented in this study reveals a trade-off between environment, health and economic development. Though the study is limited in terms of data due to the progressive nature of the COVID-19 pandemic. However, the global pandemic has demonstrated the need to empirically assess the trilemma of environment-health-economic nexus. While environmental pollution is reported to have declined due to shutdown in vital economic sectors such as transportation, aviation and industries, the health systems have been improved significantly to save lives at the expense of economic deterioration. Nevertheless, a rebound effect is projected to occur across countries via rebooting of primary economic sectors to salvage economic loss. Based on both qualitative and quantitative assessment, the post-COVID-19 is expected to rekindle and intensify environmental degradation while improving and sustaining the health system to recover and sustain economic productivity. On the contrary, the aftermath of the COVID-19 pandemic will have a long-lasting societal effect on workplaces, public places and social events, which will directly affect the economic transition. Thus, governmental effort across countries is required to strike a balance between environmental sustainability, health outcomes and sustained economic development due to the potential trade-off effects.

## Abbreviations

IMF: International monetary fund
TND: Tunisian dinar
OECD: Organisation for economic co-operation and development
RM: Malaysian Ringgit
NOK: Norwegian Krone
SARS: Severe acute respiratory syndrome-related coronavirus
WHO: World Health Organization
NASA: National Aeronautics and Space Administration

## Footnotes

1 “The WPUI index is constructed by counting the number of times uncertainty is mentioned within a proximity to a word related to pandemics in the Economist Intelligence Unit (EIU) country reports. Specifically, the index is the percentage of the word "uncertain", and its variants, that appear near the pandemic terms in EIU country reports, multiplied by 1000. A higher number means higher uncertainty related to pandemics and vice versa.” (Ahir et al. 2018).
